# Description, prediction and causation: Methodological challenges of studying child and adolescent development

**DOI:** 10.1016/j.dcn.2020.100867

**Published:** 2020-10-24

**Authors:** Ellen L. Hamaker, Jeroen D. Mulder, Marinus H. van IJzendoorn

**Affiliations:** a Methodology and Statistics, Faculty of Social and Behavioural Sciences, Utrecht University, The Netherlands; b Department of Psychology, Education and Child Studies, Erasmus University Rotterdam, The Netherlands; c School of Clinical Medicine, University of Cambridge, UK

**Keywords:** Developmental science, Causality, Directed acyclical graphs, Mendelian randomization, Target trials, Radical randomization

## Abstract

Scientific research can be categorized into: a) *descriptive research,* with the main goal to summarize characteristics of a group (or person); b) *predictive research*, with the main goal to forecast future outcomes that can be used for screening, selection, or monitoring; and c) *explanatory research*, with the main goal to understand the underlying causal mechanism, which can then be used to develop interventions. Since each goal requires different research methods in terms of design, operationalization, model building and evaluation, it should form an important basis for decisions on how to set up and execute a study. To determine the extent to which developmental research is motivated by each goal and how this aligns with the research designs that are used, we evaluated 100 publications from the *Consortium on Individual Development* (CID). This analysis shows that the match between research goal and research design is not always optimal. We discuss alternative techniques, which are not yet part of the developmental scientist’s standard toolbox, but that may help bridge some of the lurking gaps that developmental scientists encounter between their research design and their research goal. These include unsupervised and supervised machine learning, directed acyclical graphs, Mendelian randomization, and target trials.

## 1 Introduction

Research in the social and behavioral sciences can be divided into having a *descriptive*, *predictive*, or *explanatory* goal (Hernán et al., 2019; Shmueli, 2010). Each of these scientific goals requires different methods in terms of design, operationalization, model building, and model evaluation. While this may seem rather obvious at first, there is reason to assume that in practice the goal of a study is not always that clear. One particularly common situation is when researchers are actually interested in an underlying causal mechanism, but are not able to perform a randomized experiment due to ethical and/or practical limitations. The typical reaction to this problem is to avoid explicit causal language, and instead resort to less explicit statements (Grosz et al., 2020; Hamaker et al., 2015; Hernán, 2018). However, without being transparent about one’s goal, it is difficult to critically evaluate the approach that is taken, and the validity of the conclusions that are drawn based on the study; this in turn hampers scientific progress (cf. Hernán, 2018).

The aim of the current paper is to shed more light on the connection between research goals and methodology. In the first half of the paper, we begin with elaborating on the three different research goals in the context of developmental science. Subsequently, we discuss three research designs, that is, cross-sectional, longitudinal, and experimental research, and indicate how these align with the three scientific goals. As an example, we investigate to what extent published studies of the *Consortium of Individual Development* (CID; Kemner et al. this issue) fall within the categories of descriptive, predictive or explanatory research, and what research design was used. The aim of CID is to build a comprehensive model of the development of two crucial adaptive skills, that is, social competence and behavioral control, and it is based on a wide variety of data types and research designs, thereby providing a rich picture of current developmental research. In the second half of the paper, we discuss several techniques that are not yet part of the developmental scientist’s standard toolbox, but that may be used in a pursuit of one or more of the three scientific goals; these techniques include machine learning, directed acyclical graphs, Mendelian randomization, and target trials. With this review we aim to clarify the connection between research goals and research designs, and to inspire developmentalists to explore new methodological avenues to strengthen this connection.

## 2 Three goals of scientific research

Scientists are driven by different motivations, and we can classify their goals into description, prediction, and explanation (Hernán et al., 2019; Shmueli, 2010). Here we provide some examples of research questions in developmental science to illustrate the differences between these scientific goals.

When the interest is purely in describing, we need very little theory to base our research on. For instance, we may be interested in the question whether the use of social media in adolescents is related to feeling lonely. Or we may want to know whether the use of harsh punishment by the parents is positively associated with behavioral problems in children. In such cases we simply want to determine whether there is a relation between two variables, which could be captured by—for instance—a correlation coefficient or a mean difference. Typical of description is that we are not making any claims or suggestions about the origin of this association, or how this could be used to screen, select or identify individuals or individual developmental trajectories.

A second research goal is to predict a particular variable from one or more other variables. Prediction *pur sang* is based on predictors that precede the outcome in time (Hernán et al., 2019), and on an evaluation of how well the predictive model performs in out-of-sample predictions (Shmueli, 2010). Prediction allows us to identify individuals, for instance, adolescents who are at risk to become depressed, to select individuals, for instance primary schoolers who will benefit from more challenging learning materials, or to differentiate between children, for instance hyperactive children who will respond well to a more structured learning environment or to medication. The temporal order of predictors and outcome is essential to make such predictions ahead of time.

If the purpose of the study is explanation—which means we want to understand the driving forces of the underlying mechanism—we typically need to have a theory about what factors may serve as causes, and what variables are outcomes or effects of the causal process. Based on such a theory, we can develop hypotheses, such as: Excessive social media use puts adolescents at risk to feeling lonely. Or: Increased feelings of loneliness lead an adolescent to spend more time on social media. It is important to realize that the description of a causal mechanism does not always require the use of explicit causal language (Hernán, 2018); instead, critical in causal thinking is that changing *X* will result in a change in *Y*. In contrast, in descriptive or predictive thinking we may assume a higher *X* is accompanied or followed by an elevated *Y*, but there is no assumption that increasing *X* will result in a change in *Y*.

It is sometimes assumed that the goals described above form a hierarchy, where causality is the “highest” or most scientific, while the other two are mere stepping stones towards this ultimate goal (Hernán et al., 2019; Shmueli, 2010). This idea is also reinforced by the common assumption that if one understands the underlying, data generating mechanism (i.e., the causal process), this implies one can make the best possible predictions. However, this is not necessarily the case with finite samples, where sampling fluctuations may critically affect the parameter estimates of the true, data-generating model, thereby leading to less accurate predictions. For instance, Shmueli (2010) has shown analytically that in finite samples a simpler model may lead to better predictions than the true data-generating model, which further underscores the importance of being clear and unambiguous about one’s goal.

Moreover, some researchers make a distinction between causation and explanation (Hernán et al., 2019), or causal description and causal explanation (Shadish et al., 2002, p.9), where the former is simply about establishing a cause-effect relation, while the latter requires a true understanding of the actual mechanisms underlying this connection. This requires not only knowing the mediators in the cause-effect relation, but may also require knowledge of the underlying processes, which arguably could go down to the level of biochemical and physiological processes at the cell level. For most practical purposes causal description is sufficient, but detailed knowledge of the underlying processes might help to solve problems when under some circumstances manipulating the cause does not lead to its expected effect.

## 3 Research designs

When setting up a study, researchers have to decide what design they will use. We can distinguish between three broad classes of research designs, that is: a) cross-sectional research, in which a sample of cases (e.g., individuals, dyads, or families) is measured on a set of variables at one point in time; b) longitudinal research in which the same cases are measured repeatedly on the same variables; and c) experimental research, in which a putative cause is manipulated to determine its effect on a particular outcome. We discuss the strengths and weaknesses of each of these designs with respect to the three scientific goals mentioned above, and we provide a simplified summary of this—including some prominent analysis techniques—in Table 1. While some goals and designs fit more naturally together than others, we want to emphasize here not to rule out less obvious combinations. Rather, we hope the discussion here will encourage researchers to think in more detail about what they are aiming at, and how this may be achieved, hopefully a priori and as a basis for pre-registration of the research protocol at the service of enhanced replicability.

**Table 1:** Overview of the combination of the three research goals and the three main research designs, with some examples of analysis techniques, and identification of challenges.

	**Research Design**		
**Research Goal**	***Cross-sectional***	***Longitudinal***	***Experimental***
***Description***	Example techniques: • summary measures such as means, proportions, correlations; • summaries of patterns, such as principal component analysis, mixture modeling, cluster analysis	Example techniques: • latent growth curve modeling (e.g., relating intercept and slope, or slope to slope) • latent growth mixture analysis	Example techniques: • comparing performance of different existing groups • describing the relation between two outcome variables (e.g., relating brain activity and behavior in neuroscience)
***Prediction***	Less appropriate, as outcome should be situated in future An exception is when concurrent outcome is difficult or expensive to measure (e.g., screening when ultimate test is expensive or invasive)	Example techniques: • regression analysis, with outcome measured after predictors • models with lagged relations Note that often all occasions are used to estimate the model, rather than used to predict a distal outcome	Example techniques: • covariates (to account for residual variance within a condition) • using experimental outcomes as a predictor for future outcomes
***Causation***	Challenging due to potential confounding, and lack of time-order between cause and effect	Challenging due to potential confounding Example techniques: • cross-lagged panel models and Markov models that decompose observed variance into within- and between-person sources	Example techniques: • comparison of means across conditions Note that the focus may be on the effect of a non-manipulated variable on another (i.e., the mediation paradox)

### 3.1 Cross-sectional research

Cross-sectional data consist of obtaining measurements from a sample of cases (e.g., individuals, families, or dyads), at one point in time. As there is no experimental manipulation involved, cross-sectional data are also referred to as *non-experimental*, *observational* or *correlational*. These data are very appropriate to answer descriptive research questions. For instance, we may be interested in the percentage of nine-years-olds who have a mobile phone, in the difference in behavioral control between boys and girls, or whether social competence and happiness are positively related in adolescents. A limitation of cross-sectional research is that it is based on a single snapshot of people (Cattell, 1978). As a result, it is impossible to tell to what extent the individual differences that are observed reflect stable, trait-like differences between individuals, and to what extent they are the result of temporal, state-like fluctuations within individuals (Hamaker et al., 2017).

Since cross-sectional data are obtained at a single point in time, they are less appropriate for prediction, which is concerned with being able to forecast outcomes that have not (yet) been observed (Shmueli, 2010). A notable exception may be formed by scenarios where obtaining the outcome is so expensive or intrusive, that it is beneficial to be able to predict it using measures that are easier to obtain. In such cases the timing is not critical as the purpose is not forecasting, but a reduction in burden or costs. A second exception is when there is a natural time order among the variables due to their operationalization. For instance, we may have measured childhood adversities (such as having experienced physical or sexual abuse, or growing up in poverty) retrospectively, and use these to predict current adult happiness. However, it is not guaranteed that such retrospective measures of childhood adversities can replace prospective measures of childhood adversities to predict adult outcomes in the future. For example, Baldwin et al. (2019) have shown that agreement between prospective and retrospective measures of child maltreatment is poor.

When the interest is in causation, cross-sectional data need to be handled with extra caution, as most researchers are well aware. Gelman (2011) points out that causality implies a sequential chain of events, and that cross-sectional data may be helpful in ruling out particular chains of events, but that there may be multiple chains left of which one or more is true. For instance, deviant friends may lead to more deviant behaviors and vice versa. Furthermore, a widely recognized threat for causal inference are omitted variables, or confounders; such a common cause of two observed variables can lead to a spurious correlation, or a suppression of their true causal relation. Yet, while causal inferences based on cross-sectional research are difficult and some will even argue they should be avoided at all times, there are also intriguing contemporary developments in this area (e.g., Angrist and Pischke, 2009; Hernán and Robins, 2020; Imbens and Rubin, 2015; Pearl and Mackenzie, 2018), several of which will be discussed below.

It could be argued that an anomaly in cross-sectional research for studying causality is twin research, which tends to be concerned with the degree to which individual differences in a particular phenotype are caused by genetic differences, and to what extent they are caused by shared and unique environmental factors, and possibly by the interaction between these factors (Plomin, 2018). Here, the causes are not observed directly, nor are they manipulated by the researcher. Because genetic and environmental influences encompass all possible causes, there is no concern about unobserved confounding. However, the factors are extremely broad and undifferentiated, and it is unclear what is practically meant when we consider a one unit increase in the genetic or environmental factor at the individual level. Heritability represents a proportion of variance of a phenotype in a specific population at a specific time (Plomin, 2018).

Kendler and Gardner (2010) make use of a twin design that is more explicitly focused on supporting claims of causality (see also Kendler, 2008). It is based on measuring a specific environmental risk factor to which one member of a twin pair had been exposed (e.g., a major stressful early life event, or drinking before the age of 15), whereas the other twin had not had this experience, and estimating the odds of later disease (e.g., major depression or alcohol dependency). Elevated odds for the risk factor in dizygotic as well as monozygotic twins would point to a causal role of this factor (Kendler and Gardner, 2010), whereas an odds ratio of 1 for the monozygotic twins indicates the risk-disease relation can be fully ascribed to genetic factors (Kendler, 2008). Triangulation and convergence between this twin approach and another quasi-experimental method such as propensity score matching makes the causal claim more plausible (Kendler and Gardner, 2010; Ohlsson and Kendler, 2020).

### 3.2 Longitudinal research

Longitudinal data consist of two or more separate measurement occasions at which the same variables have been measured on the same cases. The vast majority of longitudinal studies in developmental research are based on panel data, consisting of a relatively small number of repeated measures (say smaller than 8), and a large number of cases. With these data, we can describe developmental trajectories over time, and investigate individual differences therein, for instance with latent growth curve modeling. Moreover, we can relate the change in one variable to the change in another, by correlating the slopes in bivariate latent growth curve modeling.

At first sight, longitudinal data may seem ideally suited for prediction, and in fact, forecasting is one of the major topics in the time series literature (Box and Jenkins, 1976; Harvey, 1989; Tuarob et al., 2017). Yet, the approach in panel research tends to differ from that in time series analysis. While many of the analyses in panel research are predicting the scores of individuals at one measurement occasion, based on their scores at the previous measurement occasion—for instance with cross-lagged relations (see Usami et al. (2019) for an overview), or Markov models (Vermunt et al., 1999)—these models are typically not evaluated in terms of how well they can forecast the future. Instead, the focus is on how well they describe the observed data across all measurement occasions. Another way in which prediction may play a role in panel research is through using person characteristics to predict a person’s trajectory (e.g., by predicting the intercept and slope in latent growth curve modeling). Yet again, this is typically done using all the data at once, rather than as a forecasting technique for unseen data.

Longitudinal research is sometimes considered as more appropriate for causal inference than cross-sectional research, as it allows for the temporal ordering of the potential cause and its outcome. Indeed, it is tempting to think of the lagged relations, which characterize many longitudinal models, as representing causal effects (Usami et al., 2019). Moreover, the possibility to control for previous levels of a variable is sometimes considered a necessity to study causality in a non-experimental setting (Allison, 2006; Bollen and Brand, 2010; Bou and Satorra, 2018). However, there are diverse ways in which this can be done, and these approaches can easily lead to different, and even conflicting conclusions when results are used for causal inference (Allison, 2006; Larzelere et al., 2010; Usami et al., 2019). Moreover, lagged relations depend critically on the distance in time between the observations (Deboeck and Preacher, 2016; Gollob and Reichardt, 1987; Ryan et al., 2018; Voelkle et al., 2012).

In recent years it has been argued that longitudinal data need to be decomposed into stable between-person differences versus temporal within-person fluctuations (cf. Hamaker et al., 2015; Berry and Willoughby, 2017). At each of these levels, different causal mechanisms are likely to operate (cf., Boker and Martin, 2018; Gische et al., 2020; Hamaker, 2012). By untangling these levels in longitudinal data, we no longer need to be concerned with unobserved stable confounders at the within-person level, or with the within-person time-varying part of unobserved confounders at the between-person level, which partly mitigates the risk of omitted variables (Hamaker and Muthén, 2020). For instance, when considering the causal effect of parental monitoring and adolescents’ behavioral problems, separating the within-family dynamics from stable between-family differences is an important step forward (Keijsers, 2016); however, there may still be time-invariant confounders at the between level (e.g., social economic status, personality factors, etc.), as well as time-varying confounders at the within level (e.g., momentary emotional well-being or openness of adolescent).

### 3.3 Experimental research

Experimental research is characterized by the fact that the researcher actively manipulates a presumed cause (i.e., the independent variable), to observe the effect this has on an outcome (i.e., the dependent variable). A particularly strong and popular experimental designs is the randomized controlled trial (RCT): It is based on randomly assigning participants to different conditions (often referred to as the treatment and control conditions), which ensures that on average there are no differences between the people that received the treatment and those who do not. Hence, random assignment rules out the threat of confounding (cf. Holland, 1986), and this is why the RCT is often considered the royal road to causal inference. Yet, there are well-known limitations associated with it, such as ethical and practical restrictions on what can be manipulated. Furthermore, there is extensive literature on the risks posed by selective drop-out and non-compliance, and how these can be mitigated (cf. Barnard et al., 1998; Sagarin et al., 2014). Moreover, while the focus in experiments is often on the causal effect of a manipulated variable at the group level, Rosenberg et al. (2018) argue that the societal relevance of lab experiments could greatly benefit from complementing this with predicting individual differences in out-of-lab behaviors. Here, we want to discuss three other considerations that put into question the ease with which the experiment-causality link tends to be made.

First, when individuals are randomly assigned to different conditions, this does not ensure that there will be no initial differences between the groups. In fact, when we use an alpha of 0.05, we should expect to find significant initial differences in one out of 20 studies, regardless of sample size.^1^ For example, in one of the randomized controlled trials of CID (L-CID; Euser et al., 2016), which was based on almost 250 families, a significant difference in symptoms of depression and anxiety was found between the two groups prior to the intervention. Such prior differences between groups are called adroitly ‘unhappy randomization’ (Wadhwa and Cook, 2019). When no pre-test is included, such a difference will go unnoticed and might inflate or deflate the estimated effect of the manipulation drastically. However, inclusion of a pre-test may also have disadvantages: For instance, it may result in sensitization of the participants to the outcome of interest, and it may lower the external validity when a pre-test is not implemented in the wide-scale application. To counter these problems, researchers may use the Solomon four-group design (Navarro and Siegel, 2018), in which half of the participants in each condition also undergoes the pre-test measure, while the other half does not. This results in a factorial design that allows for the investigation of main effects of each factor (i.e., the effect of treatment, and the effect of the pre-test), and the interaction between the two. However, the feasibility and resulting power of this design is rather low.

Second, while the internal validity of an experiment is—in principle—secured by random assignment, the external validity of an experiment depends on five different sampling specifics, which Wadhwa and Cook (2019)—building on earlier work by Cronbach (1982), who introduced the acronym *utos*—summarize with the acronym *utosti*: units of observations (*u*), treatments (*t*), outcomes (*o*), settings (*s*), and time-points (*ti*). Each experiment is based on a selection of particular sampling specifics from a population of possibilities. For instance, when considering treatments, a parent support program focusing on Videofeedback to Promote positive Parenting and Sensitive Discipline (VIPP-SD) implemented in two L-CID cohorts (Crone et al., this issue; Euser et al., 2016), is only one of a variety of interventions that could have been chosen to enhance parenting quality and help optimize child development. Similarly, when considering outcomes, the use of a prosocial cyberball game (Van der Meulen et al. this issue) forms only one facet or dimension of the complex multidimensional construct that prosocial behavior is. While the choice of treatment, type of outcome, setting (e.g. lab, school or home; Rohrer, 2018), and timing of the outcome measure is almost never object of randomization, causal inferences are often generalized across various *utosti’s* (Wadhwa and Cook, 2019).

Third, when an experiment is performed, this does not mean that all research questions are concerned with the effect of the manipulation on the outcome. For instance, the main interest may be in differences in performance between prior existing groups, such as children with a particular disorder versus children without a disorder, or different age groups (Vrijhof et al., 2016). Similarly, the focus may be on the degree to which some prior measure, such as the number of offspring or gender of the participants, predicts some measure obtained during the experiment, such as the degree to which participants’ brain activation changes when listening to a baby crying versus listening to white noise (Witteman et al., 2019).

Alternatively, the main interest may be in the relation between two outcomes of the manipulation, such as level of brain activity and behavior in response to feedback, or the degree to which the effect of the manipulation on one outcome (e.g., behavior) was mediated by another (e.g., brain activity). In L-CID (Euser et al., 2016; Crone et al., this issue) for example, families are randomly assigned to either a treatment condition (in which they receive video-feedback focused on positive parenting and sensitive discipline), or a control condition. While the primary focus of this intervention study is on whether the video-feedback changes parenting behavior, a secondary interest is the relation between neural activation of the parents (in response to distressed child facial expressions), and their parenting behavior. The latter could shed some light on the neurobiological mechanisms underlying behavioral change, which would form an important step towards bridging the brain-behavior gap. However, here we encounter what could be referred to as the *mediation paradox*: Randomly assigning participants to the experimental and control conditions (here: video feedback versus control) does not imply random assignment to the mediator (here: neural activation), and as a consequence the mediator and the outcome (here: parenting behavior) may still share unobserved common causes (for instance: personality traits), which can lead to confounder bias (cf. Cox et al., 2013; Imai et al., 2010; Shrout, 2008).

Related to this, in experimental studies on preventing less adaptive developmental trajectories and promoting optimal behavioral control and social competence the goal often is not only to show the efficacy of the intervention (e.g., a parenting support program) and to explain why the intervention works (the mediating mechanisms), but also to sort out what works for whom. However, even in randomized controlled trials the answers to such moderator questions are not entirely straightforward, because participants might not have been randomly assigned to the moderators (Bakermans-Kranenburg and Van IJzendoorn, 2015). Including an observed—rather than manipulated—moderator can provide important information about treatment effect heterogeneity or differential susceptibility to treatment (Belsky and Van IJzendoorn, 2017; Crone et al., this issue), which may be used to predict the effectiveness of treatment for different levels of the moderator (see Mayer et al. (2019) for an extension with latent moderators). However, it does not necessarily imply causal moderation, in which the moderator itself actually dampens or amplifies the effect of treatment, and could thus be manipulated to change the effect (VanderWeele, 2009). Finally, the effect of an intervention is liable to heterogeneity accruing randomly from a large variety of sources, not all of them being measured moderators. Such unavoidable treatment effect heterogeneity might jeopardize replication efforts (Kenny and Judd, 2019).

These moderator and mediator paradoxes play an often undetected role in deriving causal conclusions from experimental studies on animal (e.g., Knop et al., 2019) as well as human development (e.g., Euser et al., 2016; Crone et al., this issue). Thus, while experimental research is often considered the best approach to study causality, it does not automatically warrant high quality causal inference; the generalizability of results can be problematic, and the actual research focus may not be on the manipulation and its effect. Hence, the Pavlovian association of experiment-causality should be placed under scrutiny, and the search for alternatives of the randomized controlled trial should be intensified (Van IJzendoorn, 2019).

### 3.4 Conclusion

From the discussion above it is clear that some designs are more suitable for particular research goals than others. We have summarized the main points in Table 1, which necessarily forms a simplification of the often permeable boundaries between the different types and of the complexities of empirical research.

In particular, the large category of quasi-experimental designs (see Shadish et al., 2002), may be difficult to properly locate in this overview. Some quasi-experimental designs consist of comparing two different treatment groups, while participants were not randomly assigned to these treatments; such research designs may be considered as somewhere on a continuum between cross-sectional research and experimental research. Similarly, one may use a pre-post design, in which no control group without the intervention is used; this may be considered as some combination of longitudinal research and experimental research. To what extent one may draw causal conclusion from such quasi-experimental designs depends on additional assumptions the researchers have to make (cf. Holland, 1986; Shadish et al., 2002), and it is up to the researcher to argue why such additional assumptions are reasonable (cf. Ahern, 2018; Grosz et al., 2020; Hernán and Robins, 2020).

Researchers may also be interested in predicting certain outcomes in the distant future (e.g., health ten years from now), but it may not be practically feasible to obtain data that actually cover the entire time range; as a compromise between ideal and practice, researchers may choose to predict concurrent outcomes instead. However, when considering their ultimate goal in this scenario, researchers need to identify additional assumptions they have to make (e.g., current health is a good proxy for health ten years from now), and argue why these assumptions are reasonable.

The purpose of Table 1 is to identify what can be considered ideal combinations of research goals and research designs, and which combinations are more challenging. The latter require the researcher to make more conscious efforts to ensure a stronger connection between the two, for instance through the use of additional assumptions, or with the help of certain methodological innovations. Below, we will first evaluate the degree to which goals and designs align in the practice of empirical research, and how often more challenging scenarios are encountered. After that, we will discuss a number of methodological tools that may prove helpful in tackling common challenges.

## 4 Scientific goals and research designs in practice

To illustrate how the focus in recent developmental studies are related to the three goals that were discussed above, and what designs tend to be used, we evaluated 100 published studies.^2^ First, a random sample was drawn from the CID’s Annual Report 2017–2018, which contained an overview of all CID publications (either submitted or published) before 2019. Publications without new data analyses, such as systematic reviews, formal commentaries, essays, and review articles, were excluded from the sample, and replaced by CID papers that did meet the criteria. Second, we purposively searched for sentences regarding the research question(s), hypotheses, discussion, and conclusion of the sampled publications. We discarded sentences from coding that pertain to: a) publications other than the publication itself (e.g., “Researcher X et al. found that …”, “This is in line with findings by …”); b) strengths and limitations of the current study; c) directions for future research; and d) potential explanations for a result that was found (e.g., “This might suggest that…”, “We see two possible interpretations for…”). Third, we categorized each sampled sentence as being descriptive, predictive, or explanatory using a coding scheme (inter-coder reliability of the finalized coding scheme was .713 [Cohen’s kappa]; details can be found in the supplementary materials). We not only classified explicit causal language as falling in the category causation, but also terminology that implies causal relations, such as: affect, influence, impact, underpinnings, spill-over, risk factor, lead to, put at risk, et cetera. Such terminology refers to causation and forms a typical way to avoid explicit causal language, especially in the context of performing a non-experimental study (Grosz et al., 2020; Hernán, 2018; Pearl and Mackenzie, 2018).

**Fig. 1:**
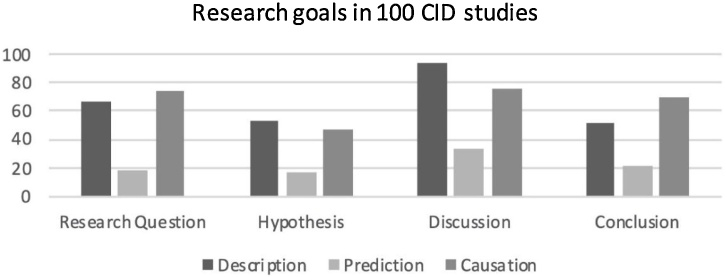
Number of papers in a random sample of 100 CID studies that indicate a descriptive/association, predictive/forecasting, or explanation/causal interest in the research question, hypotheses, discussion, and conclusion. Note that a study may include multiple goals.

In Fig. 1 we present the number of CID studies that indicate a descriptive, predictive, or causal interest in the research question, hypothesis, discussion and conclusion. Note that in each part multiple goals could be identified (i.e., out of 100 papers, 51 indicated multiple goals for the research questions, 31 for the hypothesis, 81 for the discussion, and 42 for the conclusion). It shows that CID studies are mostly driven by descriptive and causal interests. The relatively low number of studies based on a predictive goal may come as a bit of a surprise, given the interest of developmental science in predictive screening of the most vulnerable families.

**Fig. 2:**
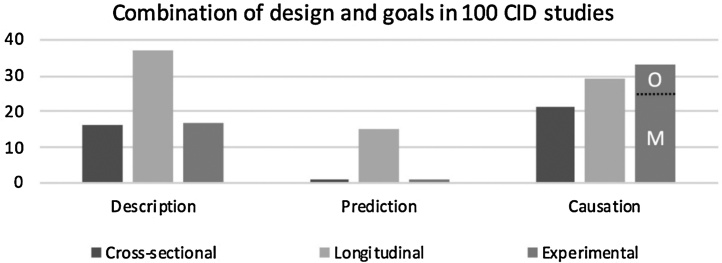
Number of papers in the random sample of 100 CID studies based on a particular design and that indicate a descriptive/association, predictive/forecasting, or explanation/causal interest in the research question. Papers may include multiple goals and multiple study designs. Experimental research focused on causation is further divided into “M” when the assumed cause X was actively manipulated by the researchers, and “O” when the assumed cause X was observed (e.g., when X is an outcome variable in the experiment, as in the mediation paradox, or a variable measured prior to the experiment; see main text for further details).

We also considered the combination of research goal and design, which is summarized in Fig. 2. It shows that a majority of studies that focus on description and/or prediction are based on a longitudinal design, whereas causal research uses an experimental design most often, although in a substantial number of cases it is also based on cross-sectional or longitudinal designs. For the experimental studies in the causal category, we made a further distinction into studies in which the assumed cause in a research question was actually manipulated versus observed (denoted as “M” and “O” respectively in Fig. 2). The latter includes: a) cases in which the assumed cause was observed prior to the manipulation; and b) research question regarding the causal effect of one outcome of the manipulation on another outcome (discussed before as the mediation paradox).

## 5 Techniques for description, prediction and causation

In this section we elaborate on various techniques that researchers can use to improve the alignment of research goals with their research design. We focus specifically on techniques that are not yet part of the standard toolbox of (neuro-)developmental scientists. Some of these techniques already have an impressive history in other disciplines such as computer science, artificial intelligence, econometrics, epidemiology, and statistics, but they are only recently becoming of practical interest to researchers in studies on human development. Because developmental researchers are often interested in explaining “why some children thrive and others do not” (e.g., CID’s overarching goal), the need for techniques that support causal inference is paramount. Therefore, we will elaborate in particular on innovative techniques to improve causal inference, but we will also discuss sophisticated techniques that can be used to improve current practice in descriptive or predictive research.

### 5.1 Techniques for descriptive research questions

Descriptive research may seem the least interesting and, as a result perhaps, the least popular of the three research goals; it is often not even mentioned as a separate goal in addition to prediction and explanation (Shmueli, 2010). Yet, our content analysis of CID studies indicates it is actually a frequent focus of developmental research. Moreover, as technology allows for increasing amounts of data to be gathered, developmental scientists are facing the challenge of how to find meaningful ways to summarize these data (Dwyer et al., 2018; Walter et al., 2019).

A promising approach to reduce the dimensionality of data is by use of *unsupervised learning*, which is part of the larger class of techniques known as machine learning (Hastie et al., 2009; Malik and Tuckfield, 2019). The general goal of unsupervised learning is to find patterns in the data. The two most common types of unsupervised learning—cluster analysis and principal component analysis—are actually quite well-known in the social and behavioral sciences. Cluster analysis can be used to obtain qualitatively different groups of individuals; for instance, Drysdale et al. (2017) used this technique to identify different neurophysiological subtypes of patients based on distinct patterns of dysfunctional connectivity in the brain. Principal component analysis has been used to study large numbers of recorded neurons, as opposed to studying neurons individually (Cunningham and Yu, 2014), and in the field of genetics it is used to study differences in genetic make-up of different populations (Patterson et al., 2006). Such unsupervised learning approaches are often used as a form of preprocessing data, to reduce the number of predictors in big data (but also in ‘small’ datasets, see Van der Meulen et al. this issue). Moreover, unsupervised learning can also be used for outlier detection (i.e., a case that does not fit the general pattern in the data).

While unsupervised learning is sometimes presented as a new field associated with big data, the techniques clearly build on the rich tradition of Exploratory Data Analysis (EDA, Tukey, 1977). Also, the concerns about a lack of replicability due to multiple testing and our human inclination to see patterns in noise—which play a lead role in the literature on machine learning in general—have actually been on the statistical agenda for decades (Diaconis, 1985; Leamer, 1978). We will discuss the problem of replicability and how to counter it, in the following paragraph and in the final section of this paper in more detail.

### 5.2 Techniques for predictive research questions

Although prediction is often considered an inferior goal in comparison to explanation, it should not be underestimated, both in terms of a research goal, but also in terms of how to accomplish it (Shmueli, 2010). As indicated above, prediction is concerned with being able to predict outcomes that have not (yet) been observed: By forecasting these scores, it becomes possible to screen individuals for early intervention, to choose the most promising intervention modality, or to match individuals with specific roles or positions. Prediction can thus be of great societal relevance.

A major threat in prediction is overfitting, which is almost guaranteed to happen when no specific actions are taken to avoid it (Polikar, 2006; Yarkoni and Westfall, 2017). Overfitting occurs when variables are selected that contribute to the prediction in the current sample, but actually worsen prediction in other samples. To illustrate the very real risk of overfitting, Raftery (1995) first randomly generated a dataset of 100 cases on 51 unrelated variables. Subsequently, he used one of these variables as the outcome variable in a regression analysis, and the other 50 variables were used as predictors. While the true *R*^2^ was thus zero, using all 50 predictors resulted in an $\hat{R^{2}}$ of 0.60. Even when using the first four predictors that explained most of the variance, $\hat{R^{2}}$ was 0.18, combined with a very small *p*-value. The adjusted *R*^2^ that is sometimes reported as a more realistic estimate of the proportion of explained variance, can also seriously overestimate how well the model will do in out-of-sample prediction (Yarkoni and Westfall, 2017). The fundamental problem here is that model performance in a particular sample is affected by the particularities of that sample, and a selected model will almost always perform less well in a different sample, even when the new sample comes from the same population (Yarkoni and Westfall, 2017).

To avoid the risk of overfitting, researchers can use cross-validation techniques (Arlot and Celisse, 2010; Dwyer et al., 2018; Mosteller and Tukey, 1977; Stone, 1974). This is based on splitting the data into a training set and a validation set (also known as the holdout or test set or sample; Polikar, 2006): The training set is used to obtain estimates of the parameters, which are then used to make predictions in the validation set. The idea behind this approach is simple and intuitive: To evaluate a predictive model we should not use the same data twice, but instead use out-of-sample metrics. Without such an evaluation, the predictive value of a model will be overestimated. There are many variations of cross-validation techniques, such as K-fold cross-validation (based on dividing the total sample into K subsamples, each of them serving as the validation set with the remaining subsamples forming the training set), and leave-p-out cross-validation (based on leaving out *p* observations at the time as the validation set, and using the remaining observations as the training set).

The concept of cross-validation lies at the heart of *supervised learning*, which is a type of machine learning that mitigates the risk of overfitting when we try to predict an observable outcome as accurately as possible (Dwyer et al., 2018; Hastie et al., 2009; Yarkoni and Westfall, 2017). The target variable can be either continuous (as in regression analysis), or categorical (as in logistic regression and nonlinear classification techniques). Cross-validation is used to evaluate the out-of-sample performance of a particular model; subsequently, alternative models can be compared on their out-of-sample performance, so that the best performing model can be selected. A nice example of this approach in the developmental neuroimaging research of CID is Qoala-T, a supervised learning tool that examines the accuracy of manual quality control of T1 imaging scans and their automated neuroanatomical labeling processed in FreeSurfer (Klapwijk et al., 2019).

Prediction may be even further improved through combining the information of multiple models, instead of selecting one model as the best model; this approach is known as ensemble methods, and includes model averaging (Burnham and Anderson, 2004), or bagging (including random forests; Shmueli, 2010). It is important to note though that, while cross-validation and supervised learning ensure that the results will replicate in other samples from the same population, they do not guarantee that results obtained from a convenience sample such as students, can be generalized to (samples taken from) another population, which may be where the actual interest lies.

### 5.3 Techniques for causal research questions

There is a vast and fast growing body of literature across diverse disciplines—including computer science, statistics, econometrics and epidemiology—that focuses on the development and promotion of innovative methodology that can improve causal inference under ideal and less ideal scenarios (e.g., Angrist and Pischke, 2009; Foster, 2010; Hernán and Robins, 2020; Pearl, 2009a, 2009b; Rosenbaum and Rubin, 1983; Schafer and Kang, 2008; Shrout et al., 2008; VanderWeele, 2015; VanSteelandt and Lange, 2012). Here we discuss three of these developments, that is, *directed acyclical graphs (DAGs)*, *Mendelian randomization*, and the *target trial*. All three fit within the more general contemporary interventionist framework, which is based on defining causality in terms of the effect a real or hypothetical intervention has on an outcome.

#### 5.3.1 Directed acyclical graphs

A causal DAG is a graphical representation of a causal structure, and consists of *nodes*, representing the variables, and *edges*, which are one-headed arrows serving as direct causal relations between nodes (Spirtes et al., 2000).^3^ A directed edge between two nodes thus represents the assumption that one variable has a direct causal effect on the other. Likewise, no edge between two nodes represents the assumption that neither variable has a (direct) causal effect on the other. DAG methodology in non-experimental research can be used in an exploratory way, to determine the causal structures that may have given rise to the observed data (Haughton et al., 2006; Sprites et al., 2000), or in a theory-driven way, to answer the question: If this DAG is the true causal structure, which other variables need to be controlled for to establish the true causal effect of *X* on *Y*? Here we focus on this latter use of DAG methodology, and in particular on its use prior to data selection, such that it can inform decisions about which variables actually need to be measured.

In this approach, researchers begin with representing their causal assumptions in a DAG, which contains *all* the variables that are believed to play a role in the causal mechanism under investigation, as well as their specific causal connections (Rohrer, 2018). Based on this DAG, we can determine whether controlling for a particular third variable *Z* will improve or actually harm the causal analysis. To this end, we first have to consider all the paths in the DAG that connect *X* and *Y* and that contain *Z*, that is, each sequence of nodes and edges between *X* and *Y* regardless of the direction of the edges. Subsequently, we need to determine whether *Z* is a *confounder*, a *mediator*, or a *collider* on each of these paths, which critically depends on the direction of the edges. Elwert and Winship (2014) argue that only confounders should be controlled for in causal analyses, while controlling for a mediator leads to overcontrol bias,^4^ and controlling for a collider is known to result in collider bias.

**Fig. 3:**
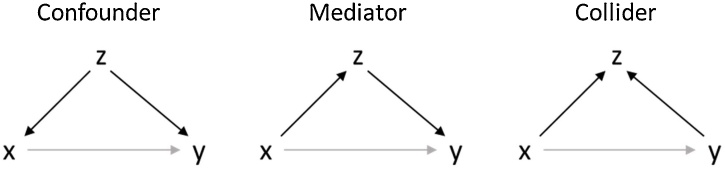
Three different directed acyclical graphs (DAGs) that show the three different roles of a third variable Z, that is: a confounder or common cause, a mediator on an indirect causal path, and a collider or effect of both variables.

Fig. 3 contains DAGs that illustrate the three different roles *Z* can have. In each DAG there are two paths from *X* to *Y*: the direct causal effect *X* → *Y*, and a second path that includes *Z*. The left panel of Fig. 3 contains the path *X* ⟵ *Z* → *Y*, where *Z* is a *common cause*, also referred to as a *confounder*. Such a path adds to the association between *X* and *Y*, and if we do not control for *Z*, this will result in confounder bias in the estimation of the causal effect of *X* on *Y*. By controlling for *Z*, we block the path X ⟵ Z → Y, a practice also referred to as *closing the back-door*. The risk of confounder bias is well recognized in psychology and related disciplines, and has in some fields led to the practice of including as many covariates as possible (Rohrer, 2018). Yet, this is not always harmless, as the other two examples show.

In the middle panel of Fig. 3 the variable *Z* is a *mediator* on the path *X* → *Z* → *Y*. Again, this path contributes to the association between *X* and *Y*, and controlling for *Z* will block this path. However, blocking this path (i.e., controlling for *Z*) implies that part of the causal effect of *X* on *Y* is removed. If the interest is in establishing the total causal effect of *X* on *Y*, one should not control for mediators, as the indirect effect through *Z* also forms part of the total causal effect. Most developmental scientists are familiar with this reasoning, and report the total, direct and indirect effects whenever there are mediators in their model.

Finally, in the right panel of Fig. 3 the variable *Z* is a *collider* on the path *X* → *Z* ⟵ *Y*, where the arrows pointing into *Z* imply *X* and *Y* causally influence *Z*. A path with a collider is by default blocked, which means it does *not* add to the association between *X* and *Y*. However, conditioning on a collider opens this path, which implies that it will now add to the association between *X* and *Y*; this is sometimes also referred to as *opening the back-door*. Since controlling for a collider (or a variable that is itself caused by a collider) biases the estimation of the causal effect of *X* on *Y*, it should be avoided at all times.

Given the major implications of controlling for a collider and its prominence in the literature in other disciplines such as epidemiology, it is somewhat surprising that this phenomenon is not very well known in developmental research. One of the few exceptions is the study by Drevin et al. (2020), who study the causal effect of childhood abuse on unplanned pregnancies in the large Norwegian Mother and Child Cohort Study (MoBa). They present a DAG (Figure 1 in their paper), which shows that the decision to have an abortion is influenced by both childhood abuse (the putative cause), and whether it was a planned or unplanned pregnancy (the outcome of interest). Since only women who did not have an abortion were selected for this study, this implies there was conditioning on (a descendent of) a collider, which will result in collider stratification bias (Drevin et al., 2020). For further elaborations, see Austin et al. (2019), who systematically discuss DAGs in the study of child maltreatment. To illustrate the role of colliders in more detail here, we provide a less complicated hypothetical example from the field of child development in Box . This shows the potential of DAGs for making more informed decisions, both in designing a study, and in distinguishing between variables that we need to control for versus variables we should actually not control for. Additionally, DAGs are also a useful tool in understanding phenomena such as selection bias, nonresponse bias, and measurement bias (e.g., Elwert and Winship, 2014; Hernán and Robins, 2020).

Box 1 **Illustration of how to use causal DAGs.**

**Fig. 4:**
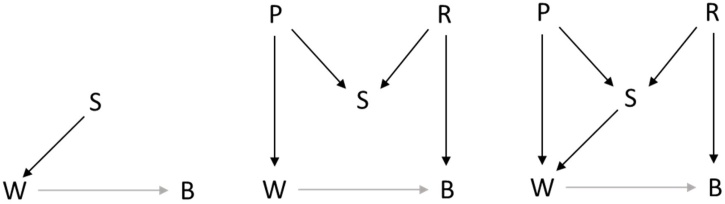
Three different directed acyclical graphs (DAGs) that show the three different roles of a third variable Z, that is: a confounder or common cause, a mediator on an indirect causal path, and a collider or effect of both variables.

Suppose there are three researchers who are interested in studying the effect of maternal warmth (W) on the child’s behavior (B), and they wonder whether there is a need to control for marital satisfaction of the mother (S). Fig. 4 contains the causal DAGs that each researcher comes up with.

The causal DAG proposed by the first researcher, presented in the left panel of Fig. 4, indicates that maternal warmth is influenced by marital satisfaction. As there is no path between treatment (W) and outcome (B), that contains marital satisfaction, controlling for the latter will not change the association between treatment and outcome.

In the middle panel the DAG of a second researcher is presented. It includes two other variables, that is the mother’s personality (P) and the relationship quality (R). The researcher argues that the mother’s marital satisfaction depends on her personality and the actual quality of the marriage. Furthermore, her personality determines her behavior and therefore the amount of warmth she shows towards her child. Moreover, the actual quality of the marriage is also a cause of the child’s behavior, as it is an important factor in shaping the larger family context and atmosphere in which the child grows up. Even though the variable P and R are not measured, a DAG that includes them can be used to determine the effect of controlling for S. To this end we need to consider the path between W and B through S, that is W⟵P→S⟵R→B. The variable S is a collider on this path, as two arrows are pointing into it. This means that this path is closed and it does not contribute to the association between W and B. However, if we condition on the collider S (e.g., by including it as a covariate), this will unblock the path, leading to bias in the estimation of the causal effect of W on B.

The third researcher comes up with a DAG that combines the ideas of the other two researchers, and is represented in the right panel of Fig. 4. In this DAG there are two paths in addition between W and B, that is, W⟵P→S⟵R→B, where S is a collider, and W⟵S⟵R→B, where R is a confounder. This implies that if we control for S, this opens the first path and introduces collider bias; however, if we do not control for S, there is confounder bias via the second path. This catch-22 is an instance of an unidentified causal effect: When only W, B and S are observed, it is impossible to obtain an unbiased estimate of the causal effect of W on B. The latter poses a difficult problem for researcher 3 who is dealing with an existing dataset; this researcher has to decide which of the two biases is expected to outweigh the other, which may be a quite impossible task. But if researcher 3 has performed this causal identification analysis prior to data collection, this researcher could conclude that only obtaining W, B, and S does not suffice, and that it is essential to include measures of either R or P. When a measure of the confounder R can be obtained, this can be controlled for in the analyses, thus blocking the path W⟵S⟵R→B; then S would not be controlled for. Alternatively, if a measure of P is obtained, then both S and P could be controlled for, where S controls for the confounder bias (as it blocks W⟵S⟵R→B), but in doing so opens the path on which S is a collider (i.e., W⟵P→S⟵R→B); controlling for P closes this path again. Moreover, when it is not possible to obtain direct measures of P or R, it may still be possible to obtain a proxy for one of them. Then, including the proxy is a way to control for part of the effect of the unmeasured variable.

Insightful follow-ups on DAGs: Glymour et al. (2005), use DAGs to decide whether one should use baseline adjustment in change analysis (see also: Pearl, 2016; Kim and Steiner, 2019; Tennant et al., 2019); Rohrer (2018) discusses DAG methodology in psychology; Lee (2012) presents an extensive account of DAG methodology in the context of personality psychology, followed by multiple commentaries from diverse disciplines, including psychology, epidemiology and computer science; Richardson et al. (2019) discuss the problem of collider bias in response to a study that considered the interaction between neuroticism and health on mortality; Glymour (2006) provides an elaborate introduction to using DAGs in the field of social epidemiology; Shrier and Platt (2008) provide a simple six-step approach that can be used to analyze a DAG to determine which variables should or should not be controlled for in the context of biomedical research; and Grosz et al. (2020) present the use of DAGs as the second step in a four-step roadmap for causal inference in psychological science.

Alt-text: Box 1

While we are convinced that familiarity with DAG methodology could prove enormously beneficial to many researchers and we have tried to make a case for it here, two critical remarks are in place. First, our coverage of the topic is necessarily brief and simplified, and does not do justice to the vast and nuanced literature that exists in this area. We hope, however, that the current treatment triggers the readers’ curiosity, and that they will use the references for follow up reading. Second, a major criticism of causal DAG methodology is that it is based on the premise that the DAG a researcher uses captures the true underlying causal structure (e.g., Imbens, 2019). How to come up with the right—or even a plausible—DAG however, is a major challenge. As Rohrer (2018) states, it is not a mechanistic procedure, but requires assumptions and thorough domain knowledge. While this can be seen as a major drawback of DAG methodology, the advantage is that it forces researchers to be concrete about their assumptions, which facilitates communication, transparency, and discussion. The call for pre-registration of study protocols might be addressed by detailed discussions about the merits and plausibility of DAGs involved before the study starts.

#### 5.3.2 Mendelian randomization

A promising approach to handle unobserved confounding in non-experimental research is through Mendelian randomization, which can be considered a special case of the more general technique of using *instrumental variables.* The latter is a particularly popular approach for studying causality in non-experimental studies in the field of economics (Angrist and Pischke, 2009), although there are also major challenges associated with this technique, most notably the difficulty of finding a good instrument (cf. Angrist et al., 1996; Hernán and Robins, 2020; Stock and Yogo, 2005). Here, we will explain the basics of using instrumental variables first, and then describe how genetic variables have been used as instruments to avoid unobserved confounder bias.

**Fig. 5:**
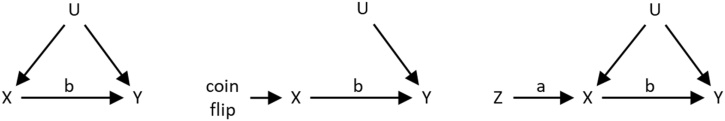
Three directed acyclical graphs (DAGs). Left DAG shows an observational study in which the causal effect of X on Y is biased due to unobserved confounding U. Middle DAG shows how randomizing treatment (through a flip of a coin), breaks the causal effect of U on X, thereby allowing for the estimation of the causal effect of X on Y. Right DAG shows the use of an instrumental variable Z in a non-experimental study: By dividing the effect of Z on Y (a*b) by the effect of Z on X (a), one can estimate the unbiased effect of X on Y (b).

Suppose we are interested in the effect of Ritalin (*X*) on attention deficit hyperactivity disorder (ADHD) symptomatology (*Y*). As shown in the left panel of Fig. 5, if we simply observe *X* and *Y*, the relation is likely to be confounded for instance by the severity of the disorder (*U*), as children with more severe forms of ADHD are more likely to receive medication than children with less severer forms. To obtain an unbiased effect of *X* on *Y*, we could perform a randomized controlled trial (RCT) in which children are assigned to receive medication or not based on the flip of a coin. This is depicted in the middle panel of Fig. 5, which shows that if treatment *X* is completely determined by random assignment, it no longer shares the confounder with the outcome *Y*. However, when an RCT is not feasible, we can search for an instrumental variable *Z*, which has to satisfy the following three conditions (Davies et al., 2018; Hernán and Robins, 2020). First, the instrument should be related to the cause *X,* and this relation should not be too weak; note that this can be tested in practice. Second, the instrument should not share any common causes with the outcome *Y*. Three, the instrument should affect *Y* only through *X*. The latter two conditions cannot be tested, and have to be argued based on domain knowledge. In the current example, we could consider the parents’ attitude towards psychoactive drugs as an instrumental variable, as: 1) this is likely to have an effect on whether or not parents decide to give their child Ritalin (i.e., *X*), and this should be tested; 2) we may assume it does not share common causes with the ADHD symptomatology (i.e., *Y*);^5^ and 3) it is also unlikely to affect the ADHD symptomatology through anything else but the treatment (i.e., *X*). Here, we first estimate the total effect of *Z* on *Y*; this is identical to the indirect effect through *X* in this model, which—in the linear case—is the product a*b. Subsequently, we estimate the effect of *Z* on *X*, which is *a*. Then, by dividing the first by the second, we obtain an unbiased estimate of *b*.

In Mendelian experiments, which are increasingly used in epidemiology, the instrumental variable may be either a Single Nucleotide Polymorphism (or SNP, i.e., allelic variation in a single nucleotide in DNA; Van IJzendoorn, 2019), or a (dichotomized) version of a Polygenic Risk Score (PRS), which consist of thousands of SNPs (Plomin, 2018). Such a genotype score serves as a genetic coin flip dividing the sample already at conception into a ‘treatment’ group at risk for a specific exposure or phenotype, and a ‘control’ group not at risk for this exposure or phenotype, randomly distributing all other potentially confounding factors (only if pleiotropy is absent or controlled for; see Ference, 2018). While in principle the genotype measure should only affect the outcome indirectly through its effect on the exposure of interest (Davies et al., 2018), it is also possible to block additional indirect (genetic) paths by conditioning on mediators on such paths.

**Fig. 6:**
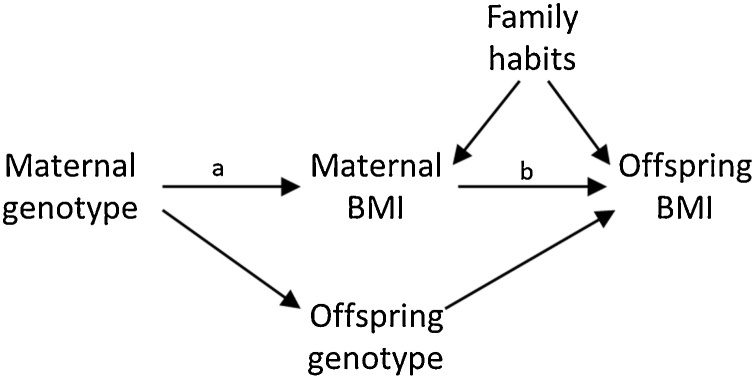
DAG for intergenerational Mendelian experiment for investigating the causal in uterus effect of maternal BMI on future offspring BMI, with family eating habits as unobserved confounders, maternal genotype as the instrumental variable, and offspring genotype as mediator that needs to be controlled for. Based on Figure 1 in Richmond et al. (2017).

To give an example of this, Richmond et al. (2017) performed an intergenerational Mendelian experiment on data from Generation R—a cohort study which is part of the CID—to test the hypothesis that maternal pregnancy obesity causes future offspring obesity. Specifically, they hypothesized that maternal prenatal body mass index (BMI) affects the BMI of children through birth weight, but also through critically affecting appetite control, neuroendocrine functioning, and /or energy metabolism of the child. As represented in Fig. 6, the relation between maternal BMI and child BMI is of course likely to be confounded by habits of the family regarding food and exercise, *and* by genetic factors that the mother and child share. In this scenario, the genotype score for maternal BMI can be used as an instrumental variable based on: a) the assumption that it is unrelated to the environmental common causes of the maternal BMI and offspring BMI relation (pleiotropy should be absent, i.e., the BMI genotype is not related to other phenotypes that may affect offspring BMI); and b) the inclusion of the offspring genotype score for BMI to block the second indirect effect from the instrumental variable (Maternal genotype) to the outcome (Offspring BMI). In that case, the effect of maternal genotype on offspring BMI will be the indirect effect, only mediated by maternal BMI (i.e., a*b). Dividing this by the effect of maternal genotype on maternal BMI (i.e., *a*), gives an estimate of the effect of maternal BMI on offspring BMI (i.e., *b*).

Davies et al. (2018) discuss several tests that can be run to see whether the underlying assumptions are violated, and they describe how to report on the analyses. Particularly important is that an instrumental variable explains a reasonable proportion of the variance in the cause *X*. Stock and Yogo (2005) provide substantiated guidelines on how to decide whether an instrument is “good enough”. For BMI and for educational achievement, PRSs have been developed that are rather strongly related to the phenotypes, with explained variance of around 15%–20%, and there is great optimism that these percentages will go up in the near future with better assessment of the whole genome (Plomin, 2018). Hence, we can expect that Mendelian experiments will become increasingly popular in the near future to study causality when a randomized controlled trial is not feasible.

#### 5.3.3 The target trial

As indicated above, contemporary approaches to causality are based on defining causality as the effect of an intervention. In this context, a number of assumptions need to hold in order to draw causal conclusions (Hernán and Robins, 2020; Rosenbaum and Rubin, 1983). Three key assumptions are: 1) *exchangeability*, which means that receiving the treatment or control condition depends only on the measured covariates (i.e., there are no unobserved confounders); 2) *positivity*, that is, for each combination of the covariates that are needed to ensure exchangeability, the probability of being assigned to treatment or no treatment needs to be larger than zero; and 3) *consistency*, that is, the treatment values under investigation should correspond unambiguously with a well-defined intervention.

When focusing on this third assumption, we can see this actually has major consequences for our thinking about causality. It implies that if we are interested in the effect of stress on well-being, it is not enough to distinguish between stressed and non-stressed conditions; instead, we have to be very specific about *what kind of stress* we are considering, because being stressed about an upcoming test may have a different effect on well-being than the stress due to having a conflict with one’s parents, going on a first date, or being bullied at school. When there are different ways in which we can lift the treatment score from zero to one, this implies there are multiple versions of treatment (i.e., a population of *t*’s within *utosti*, see Wadhwa and Cook, 2019), which in turn means that there is not a single treatment effect and it becomes unclear what causal effect we are trying to study.

To counter this problem, Hernán and Robins (2020) propose the use of a *target trial*, a hypothetical intervention study that one would perform if there were no ethical and/or practical barriers (cf. Dorn, 1953). Hence, the point of specifying the target trial is not to actually execute it, but to clearly articulate the causal question we try to answer. The target trial should state in an unambiguous way: a) how the treatment variable is raised by one unit, such that it is completely clear how the treatment condition differs from the non-treatment condition (*utosti*’s *t*); and b) what the outcome is (*utosti*’s *o*), including at what point in time or during what time interval after the treatment the outcome is measured (*utosti*’s *ti*). The process of formulating the target trial is likely to consist of having to rephrase and refine the research question multiple times; Hernán and Robins (2020) indicate that this iterative process and the discussion with colleagues on what exactly the causal question of interest is, should be considered an inextricable part of causal inference research. Note that this fits perfectly to the concept of (pre-)registration of study designs, which also requires detailed descriptions of primary hypotheses, how they are tested and whether confounders play a role (Van’t Veer and Giner-Sorolla, 2016).

While the concept of a target trial has been primarily proposed as a way to determine how to best analyze large cross-sectional datasets to support causal inference, it can also be used in longitudinal research to explain the nature of within-person versus between-person differences. For instance, Dietvorst et al. (2018) considered the relation between adolescents’ secrecy and parental privacy invasive behaviors (perceived by the adolescents) in longitudinal data. Using a statistical model that separates stable between-person differences from the within-person dynamics (Hamaker et al., 2015), they found that at the between-person level there was a positive relation, whereas at the within-person level there was a negative lagged relation from secrecy to privacy invasion. When evaluating such results, it is important to realize that the stable between-person differences in the data are the cumulative results of prior within-person causal processes that differed across individuals (cf. Baltes et al., 1988). Hence, within-person fluctuations in the data are by definition associated with causal processes that take place at a shorter time-scale than the causal processes that result in stable between-person differences in the data. This implies that a target trial that is related to the within-person results probably consists of a short-term or momentary intervention (e.g., a temporary increase in secrecy), whereas a target trial that is related to the between-person results is most likely to consist of a prolonged intervention that takes place over a particular period of time, typically longer than the interval spanned by the study. Disentangling the short-term, within-person dynamics from the long-term, stable between-person differences, and linking these results to different target trials, forms a first step in uncovering the complexity of multiple processes operating at different timescales in individual development (see also Driver and Voelkle, 2018).

#### 5.3.4 In conclusion

Due to space limitations, we are not able to discuss the ins and outs of causal inference research in more detail. A more comprehensive treatment of this topic would include a discussion of the actual act of estimating the causal effect (e.g., through the use of techniques such as inverse probability weighting, matching, or g-estimation), and a discussion of the ways researchers can test the assumptions on which their causal model is based (such as sensitivity analysis (e.g., Hernán and Robins, 2020), and local (mis)fit analysis (e.g., Shipley, 2003; Thoemmes et al., 2018)). For a more elaborate discussion of the diverse steps in causal research, we refer the reader to the recent work of Grosz et al. (2020; see also Ahern, 2018; Hernán and Robins, 2020).

## 6 Discussion

We have argued that the research goal should be leading in decisions on what design, techniques, and assumptions are needed in its pursuit. Hence, being unambiguous and explicit about one’s goal and how this aligns with the design that one plans to use, is a critical step that has perhaps been skipped over too easily in practice. While certain goal-design combinations—such as a causal goal with a cross-sectional design—are widely recognized as challenging, others—such as prediction-longitudinal or causation-experiment—tend to be considered as ideal. However, we caution against over-simplifications either way. On the one hand, some combinations may be less ideal, but nevertheless the only practical possibility. An open discussion about the additional assumptions that are needed to align the design and goal under these circumstances will be more beneficial than upfront dismissing a particular combination. On the other hand, seemingly ideal combinations should not be taken for granted. For instance, the use of a longitudinal design does not automatically fit with a focus on prediction, as the latter requires an evaluation out-of-sample performance. Moreover, the real interest in an experimental study may actually be on the effect of a cause that was itself not manipulated. We hope the discussion of these issues forms an invitation to be (even) more deliberate about one’s research goals, and to consider more consciously how these connect to the research design researchers (plan to) use.

There are two closely related concerns, which are fundamental to scientific research, but that were only touched upon in passing so far: Replicability and generalizability. Here we offer a brief discussion of each, with a specific focus on how these concerns relate to some of the main themes of the current paper.

### 6.1 Replicability

It has been argued that the lack of replicability of empirical results may well be rooted in the fact that experiments tend to focus on a very narrow part of the method space, holding as many factors fixed as possible (Baribault et al., 2018; Elliott et al., 2020; Heckendorf et al., 2019). To counter this problem, Baribault et al. (2018) propose *radical randomization*, which consists of a combination of many micro-experiments that differ from each other in particular facets—such as the exact stimuli (*t*), or setting (*s*)—that could serve as moderators of the treatment effect. While each micro-experiment itself is underpowered (due to small sample size), combining them in a multilevel model allows for the direct investigation of the robustness of a particular effect against variations in *utosti* (see also Kenny and Judd, 2019). A prospective instead of post hoc replication approach might be needed to systematically vary one potential design assumption or feature at a time (Steiner et al., 2019). A related approach is *individual participant data* (IPD) meta-analysis, which originated in biomedical research (Riley et al., 2010), and is now slowly gaining ground in developmental science (e.g., Roisman and Van IJzendoorn, 2018; see Gardner et al., 2018 for an application to the Incredible Years interventions to promote children’s prosocial behaviour). It is based on combining the raw data from individual participants across multiple studies in a multilevel model. This approach is more powerful than conventional meta-analysis (which uses study summaries instead of raw data), and it allows for the inclusion of moderators not only at the study level, but also at the individual level, thus providing a richer picture of the effect and its individual differences.

Both radical randomization and IPD meta-analyses emphasize the importance of using a large number of studies with slightly varying methods and designs to obtain robust and replicable results (Van IJzendoorn, 1994). A related issue is that choices made during pre-processing the raw data may critically affect the results of the final analysis. Botvinik-Nezer et al. (2020) asked 70 independent teams to analyse the same neuroimaging dataset and found no two teams applying the same analytic strategy, resulting in alarmingly large variability in reported findings. To combat the arbitrariness of results arising from a multitude of subjective decisions, Steegen et al. (2016) propose *multiverse analysis*, which is based on considering all the different reasonable choices for pre-processing the raw data, and analyzing all the datasets that result from this. Moreover, publication bias against null results (Rosenthal, 1979), and the profuse use of ‘researcher degrees of freedom’ (see Luck and Gaspelin, 2017 for an example from EEG research), might be counteracted by (pre-)registration (Kvarven et al., 2019). Yet, it should also be noted that even robust and replicable results may be of little value if they are based on statistical models that are not properly linked to an underlying theory (Larzelere et al., 2010; Oberauer and Lewandowsky, 2019; Szollosi et al., 2019).

Clearly, the machine learning techniques, cross-validation, and ensemble methods which we discussed, are specifically designed to counter the problem of non-replicability. Yet, while some of the other techniques we discussed—such as DAGs, Mendelian randomization, and the target trial—are not specifically concerned with replicability, they may indirectly support replicability by requiring careful a priori reflection and debate on design and analytical strategies. Ideally, this will lead to decisions made in advance, which result in better documented pre-registration of experimental as well as non-experimental studies, which decrease the researcher’s degrees of freedom and thereby promote replicability.

### 6.2 Generalizability

It is often argued that the ultimate goal of science is to uncover general laws (cf. Hamaker, 2012). While finding replicable results may be seen as an important step towards this ultimate goal, there is also a large body of literature arguing that averages and aggregates obtained at the level of the population cannot be automatically generalized to the individual (cf. Molenaar, 2004).

The notion that individuals may differ, is actually fundamental to much of the causal literature, which is based on defining the individual causal effect as the difference between two possible outcomes for a particular individual: the potential outcome when treated versus the potential outcome when not treated. This difference is in essence person-specific: While some individuals benefit from treatment, others may worsen because of it, while yet others remain unaffected. However, as it is fundamentally impossible to observe both potential outcomes for a given person at the same time (cf. Holland, 1978), researchers have to resign to alternatives, such as estimating the average causal effect, which averages over the individual causal effects of individuals, or the average causal effect of the treated, which averages over the individual effects of individuals who (are like those who) were treated.

It is tempting to assume that such an average causal effect represents the causal effect for each individual; yet this requires the additional assumption of a constant effect (Holland, 1978). In a similar vein, predictive and descriptive results obtained in a sample of individuals represent averages and are not necessarily generalizable to any particular individual (Hamaker, 2012; Molenaar, 2004). To obtain results that pertain to a specific individual, it may be necessary to study that individual in depth over time, such as described by Tuarob et al. (2017) in the context of forecasting, or by Holland (1978) who proposes the assumptions of temporal stability and causal transience to allow for the study of causality in an individual (Kratochwill et al., 2010). This may result in idiographic descriptive, predictive, and causal results, which are hard to generalize to the population. However, if this is a concern, one should also realize that generalization is a two-way street here: If it is not possible to generalize from the individual to the population, it is also impossible to generalize from the population to the individual. Simply using a technique that averages over individuals does not guarantee the discovery of general laws that apply to each and every individual (or even a single individual; cf. Hamaker, 2012).

### 6.3 Conclusion

In our study based on 100 CID studies we have found that researchers often pursue multiple goals, and that their design not always aligns easily with their goal(s). It should be noted though that the current sample of papers is not a random sample from developmental research in general; to study the latter it may prove fruitful to repeat this exercise with a random selection of studies published in the most influential developmental journals instead.

To conclude, it is important to note that one goal is not higher or more scientific than another; rather, description, prediction and explanation should be considered as complementary components of science, each with their own strengths, challenges, and purpose. Description can be useful to establish which phenomena may be of interest to predict or explain, and it can be considered critical for laying down the groundwork from which researchers can develop new theories. Prediction is useful to identify *who* needs an intervention most or adapts optimally to a particular role or niche in life. Explanation is necessary to decide *how* to intervene or to understand *why* someone adapts well. This implies that preventive, clinical, and policy applications should be the outcome of cumulative slow science (Van IJzendoorn, 2019), and that results from single studies should always be evaluated within the broader context of descriptive, predictive and explanatory concepts and findings within developmental research.

## Declaration of Competing Interest

The authors declare that they have no known competing financial interests or personal relationships that could have appeared to influence the work reported in this paper.

## Footnotes

1 While sample size does not mitigate the risk of significant differences occurring, it does imply that in general the size of the differences will be smaller to the point that they become irrelevant.

2 For details, see the supplementary materials on: https://github.com/JeroenDMulder/CID-goals-and-design

3 Readers familiar with structural equation modeling (SEM) may notice a resemblance between DAGs and SEM models. However, important differences are that: a) typically, a DAG does not contain two-headed arrows to represent covariance, but will instead contain a (possibly unobserved/unobservable) common cause to account for covariance (although semi-Markovian DAGs form an exception; see Pearl, 2009b); b) a DAG typically does not contain nodes that represent random measurement error terms; c) an arrow in a DAG is a qualitative statement that there is a direct causal connection, but it does not involve information about the strength or even the shape of this relation; and d) a DAG cannot include feedback loops, meaning that if we follow a sequence of arrows starting at variable X, we can never return to X (hence the term acyclical).

4 An exception is formed by the front-door criterion, discussed by Pearl (2009b), when there is unobserved confounding for *X* and *Y*, and there is a mediator *Z* that is not directly affected by the confounder. In this case, the average total effect of *X* on *Y* can be estimated by conditioning on the mediator.

5 Some may argue that the severity of the underlying disorder *U* is likely to affect the attitude of parents towards psychoactive drugs *Z*; in that case, the latter cannot be used as an instrumental variable. This illustrates the difficulty associated with finding an appropriate instrument in practice.
